# Risk Assessment for Malignant Transformation in Patients with Oral Proliferative Leukoplakia: A 10-Year Retrospective Cohort Study

**DOI:** 10.3390/cancers18010002

**Published:** 2025-12-19

**Authors:** Gianluca Tenore, Ahmed Mohsen, Paolo Junior Fantozzi, Andrea Golrang, Gian Marco Podda, Federica Rocchetti, Lucia Borghetti, Laura Sansotta, Cira Rosaria Tiziana Di Gioia, Umberto Romeo

**Affiliations:** 1 Department of Oral and Maxillofacial Sciences (SOMF), Sapienza University of Rome, 00161 Rome, Italy; gianluca.tenore@uniroma1.it (G.T.); paolojunior.fantozzi@uniroma1.it (P.J.F.); golrang.1740483@studenti.uniroma1.it (A.G.); gianmarco.podda@uniroma1.it (G.M.P.); federica.rocchetti@uniroma1.it (F.R.); lucia.borghetti@uniroma1.it (L.B.); sansotta.2041402@studenti.uniroma1.it (L.S.); umberto.romeo@uniroma1.it (U.R.); 2 Department of Radiological, Oncological and Pathological Sciences, Sapienza University of Rome, 00161 Rome, Italy; cira.digioia@uniroma1.it

**Keywords:** leukoplakia, malignant transformation, oral cancer, oral squamous cell carcinoma, proliferative leukoplakia, proliferative verrucous leukoplakia

## Abstract

**Objectives**: This study aimed to evaluate the malignant transformation (MT) risk profile in patients with oral proliferative leukoplakia (OPL) referred to the Oral Medicine and Oral Surgery units of Umberto I Hospital, Sapienza University of Rome. **Methods**: The departmental database and medical records of OPL patients were reviewed from January 2014 to June 2024. Demographic, clinical, and histopathological features and treatment strategies were collected in a de-identified dataset. **Results**: A total of 51 OPL patients (33 females and 18 males; mean age 62.86 ± 13.55 years) were included. MT occurred in 17.6% (*n* = 9) after a mean follow-up of 4.78 ± 2.59 years. A higher percentage of the presence of a previous history of solid or hematological tumor was observed in patients with MT, with an OR of 2.940 (95% CI 0.064–1.350), without statistical significance. The homogeneous clinical form was more common in patients without MT (78.57%), and the speckled clinical form was more common in patients with MT (44.44%). The percentage of patients with lesions located on the floor of the mouth, ventral surface of the tongue, and dorsal surface of the tongue was higher in patients with MT. The tongue was the most common site of MT, followed by the gingiva, buccal mucosa, and palate. At the histological level, a verrucous, nodular, or bulky architecture was more commonly observed in patients with MT, and the presence of band-like lymphocytic infiltrate was observed in all patients with MT. Higher dysplasia grades were significantly associated with MT (*p* = 0.009). No significant association was found between the treatment modality and MT risk, although laser ablation was associated with a trend toward lower risk. **Conclusions**: This study further suggests that the clinical morphology, lesion site, and histological grading may be important predicting factors for MT in OPL. The presence of a non-homogeneous lesion form, a higher grade of dysplasia, and a history of previous solid or hematological tumor led to a more aggressive disease course. Individualized risk assessment and long-term surveillance may be advisable.

## Simple Summary

Oral proliferative leukoplakia is an uncommon and highly aggressive disorder of the oral mucosa, characterized by multifocal involvement and a substantial risk for malignant transformation. The prognostic factors remain poorly defined, which limits the ability to accurately identify patients at the highest risk. In this study, a ten-year cohort of patients was retrospectively reviewed to delineate the clinical and histopathological characteristics associated with the progression to oral cancer. The lesion morphology, anatomical site, and severity of epithelial dysplasia may be important predicting factors for malignant transformation. Homogeneous lesions demonstrated a lower risk, while speckled/verrucous forms and high-grade dysplastic lesions showed increased risk. These findings further suggest the aggressive biological behavior of oral proliferative leukoplakia and emphasize the importance of individualized risk stratification and long-term surveillance to guide clinical management and early intervention.

## 1. Introduction

Oral leukoplakia (OL) is defined by the World Health Organization (WHO) as “a predominantly white plaque of questionable risk having excluded (other) known diseases or disorders that carry no increased risk for cancer”. It is considered the most important potentially malignant oral condition, with a worldwide prevalence of about 1.4% and an overall malignant transformation (MT) rate of 6.64% [1,2].

Oral proliferative leukoplakia (OPL) is a rare aggressive subtype of OL, characterized by a progressive and irreversible disease course. It clinically presents with multiple oral leukoplakia lesions with a higher prevalence among non-smoking females in their sixth decade, with no racial predilection. The most commonly affected sites are the gingiva, buccal mucosa, and tongue [3,4].

The clinical presentation of OPL may vary. It usually manifests as multisite nodular or verruciform white plaques. However, it can also show clinical and histopathological features similar to oral lichen planus, including white striations and lichenoid or lymphocytic infiltrate and basal-cell degeneration [5,6,7].

Unlike OL, the etiology and progression to MT of OPL remain largely unknown, with reported MT rates reaching as high as 61.0% over an average follow-up of 7.4 years and an annual MT rate of approximately 10% [5,8]. Previous studies have suggested that the progression of OPL towards malignancy is mainly linked to clinical, histopathological, and molecular gene alterations. These include, but are not limited to, mutations in TP53, FAT1, NOTCH1, and p16INKA as well as the loss of heterozygosity at chromosome arms 3p, 9p, and 17p [5,9,10]. These findings indicate that OPL possibly evolves through a multistep process, where successive molecular and structural abnormalities gradually shift the tissue towards a dysplastic or malignant phenotype.

Histopathological findings from initial biopsies may also be crucial in predicting the MT of OPL. Patients with oral epithelial dysplasia (OED) at first biopsy demonstrated a significantly higher risk of transformation (ranging between 25% and 97%) compared to those with non-reactive hyperkeratosis/parakeratosis (HkNR), which exhibit MT rates between 12% and 50% [5,11].

Epigenetic alterations have also been implicated in the pathogenesis of OPL. The upregulation of oncogenic microRNAs (miRNAs) and downregulation of tumor-suppressor miRNAs have been shown to have a significant influence on the MT of OPL [12,13]. However, reliable biomarkers capable of predicting progression on an individual basis are lacking, despite advances in understanding these pathways. This has made the clinical decision-making process for OPL particularly complex.

A recent systematic review evaluated the current evidence on the MT of OL, including all relevant studies published worldwide [1]. The authors noted that only a small number of primary-level studies provided longitudinal data, whereas most available studies presented aggregated findings that limited detailed subgroup analyses of demographic and clinicopathological characteristics. They recommended conducting future research that reports individual participant data to enable a more comprehensive assessment of the factors influencing the MT of OL.

This study aimed to share our experience and evaluate the risk profile of patients diagnosed with OPL who were referred to the Oral Medicine and Oral Surgery units of the university hospital over the past ten years. We examined the impact of various demographic, clinical, histological, and management strategy-related variables on the MT.

## 2. Materials and Methods

A hospital-based retrospective single-center cohort study was conducted on adult patients with a diagnosis of OPL referred to the Oral Medicine and Oral Surgery units of the Department of Oral and Maxillofacial Sciences (SOMF), Umberto I University Hospital of Sapienza University, Rome. The included patients provided informed consent for research participation. The approval for the study was obtained from the departmental review board (Prot.nr. 0001762, 1 October 2025). All the study procedures adhered to the ethical principles outlined by the institutional and/or national research committee and complied with the 1964 Helsinki Declaration and its later amendments or equivalent ethical guidelines. All clinical and histopathological assessments were performed according to standardized diagnostic and follow-up protocols routinely used in the Oral Medicine and Oral Surgery units.

### 2.1. Inclusion and Exclusion Criteria

All OPL patients were diagnosed based on the criteria established by Villa et al. [5,8] (Supplementary Table S1). The department database and medical records were reviewed between January 2014 and June 2024. The inclusion criteria were OPL patients with (1) detailed photographic documentation of the oral lesions, (2) a complete histopathological report confirming the microscopic features consistent with the diagnosis of OPL, and (3) follow-up of at least 12 months to document any clinical progression of the lesions. The exclusion criteria were OPL patients with (1) irregularly documented clinical follow-up for at least 12 months from the first visit; (2) lack of complete photographic documentation of the lesions; (3) clinical and/or histological evidence compatible with reactive lesions (e.g., the presence of mechanical trauma, chronic hyperplastic candidiasis, or benign alveolar ridge keratosis); and/or (4) age under 18 years old.

### 2.2. Data Collection

The collected data were divided into four categories: (1) demographic and anamnestic data, (2) clinical characteristics, (3) histological characteristics, and (4) management strategies. For demographic and anamnestic data, the collected data were age, sex, tobacco status and consumption amount, alcohol status, solid or hematological tumor history, and any other concomitant diseases. For the clinical characteristics, the number and anatomical location of the lesions were documented. The clinical pattern was classified into four forms: homogeneous, verrucous, nodular, and/or speckled. The presence and number of these forms were recorded for each included patient.

There were four main histological characteristics considered: HkNR, epithelial hyperplasia, band-like lymphocytic infiltrate, and/or verrucous, nodular, or bulky architecture. For each patient, the presence and number of these features were documented. In cases where dysplastic changes were observed, the highest grade of OED (low, moderate, or severe), the evidence of MT—i.e., the development of oral squamous cell carcinoma (OSCC) or oral verrucous carcinoma (OVC)—the histological grading (in situ, G1, G2, G3), and the anatomical site of development (coded according to the 11th edition of the International Classification of Diseases (ICD-11; 2019/2021 version)) were recorded [14]. The management strategy was recorded for each patient, classified into two categories: conservative management (i.e., “watch and wait”) and surgical treatment. For each patient, the number of follow-up visits and the total number of performed biopsies were recorded.

The prognostic assessment for patients managed either surgically or with a conservative “watch-and-wait” approach was based on longitudinal clinical follow-up, documentation of the treatment modality (including excision biopsy, laser ablation, or combined procedures), and histopathological grading of any malignant transformation observed during the study period.

Patients lacking essential clinical or histological data required for outcome assessment were excluded according to the predefined criteria; no imputation for missing data was performed.

### 2.3. Statistical Analysis

All the collected data were recorded in an Excel spreadsheet (Office 365; Microsoft Corporation, Redmond, WA, USA) and were analyzed with Jamovi statistical software version 2.3.28. Descriptive statistics were calculated for all study variables. The total sample was stratified into two groups based on the presence or absence of MT. Associations between MT and categorical variables were assessed using the chi-square test or Fisher’s exact test, as appropriate. For continuous variables, independent-samples t-tests were applied, and the Mann–Whitney U test was used for non-normally distributed metric data. Adjusted odds ratios (ORs) with 95% confidence intervals (95% CI) were computed for all associations. Statistical significance was defined as *p* ≤ 0.05.

## 3. Results

A total of 51 OPL patients met the inclusion criteria with an average age of 62.86 ± 13.55 years. The majority of patients (62.75%) were over 60 years old. The major part of them were non-smokers (41.18%) and non-drinkers (94.12%). A high proportion had concomitant systemic diseases (76.47%), with cardiovascular diseases being the most common, followed by thyroid disorders and osteoporosis. Twelve patients had a prior history of solid malignancy. Patients were followed up for a mean duration of 4.47 ± 2.45 years, with an average of 4.82 ± 1.80 clinical visits per year. A mean of 3.98 ± 2.86 biopsies was performed per patient, corresponding to an average of 1.05 ± 0.89 biopsies per year.

### 3.1. Demographic and Anamnestic Data

With respect to patient-related factors, a higher percentage of the presence of a previous history of solid or hematological tumor was observed in patients with MT with an OR of 2.940 (95% CI 0.064–1.350) but without statistical significance; there were two patients with a history of oral cancer, one patient with a history of skin cancer, and one patient with a history of esophageal cancer. In addition, a high tobacco consumption in ex-smokers was observed in patients with MT (average of 29.70 ± 28.30 pack years), while in patients without MT, the average was 13.60 ± 12.20 pack years (Table 1). Regarding the evaluation of the additive or synergistic effect of smoking and alcohol consumption, two patients were drinkers: one had a history of both smoking and drinking, while the other had a history of alcohol consumption only; neither patient had MT. A full analysis of the additive or synergistic effects between these variables could not be performed, since the data related to former drinking habits were not consistently available in the clinical records.

**Table 1:** Oral Proliferative Leukoplakia (OPL): Demographic and Risk of Malignant Transformation (MT).

Variables	Total (*n* = 51)		Without MT (*n* = 42)		With MT (*n* = 9)		*p*-Value
**Age:** average (SD)	62.86 (13.55)		62.52 (14.23)		64.44 (10.27)		0.729
≤60	19 (37.25)		17 (40.48)		2 (22.22)		0.455
>60	32 (62.75)		25 (59.52)		7 (77.78)		
**Gender:** *n* (%)							
Female	33 (64.71)		28 (66.67)		5 (55.56)		0.703
Male	18 (35.29)		14 (33.33)		4 (44.44)		
**Tobacco status:** *n* (%)							0.855
Non-smokers	21 (41.18)		18 (42.86)		3 (33.33)		0.720
Ex-smokers	14 (27.45)		11 (26.19)		3 (33.33)		0.664
Smokers	16 (31.37)		13 (30.95)		3 (33.33)		1.000
**Tobacco consumption amount (pack years):**							
Ex-smokers: average (SD)	16.80 (16.60)		13.60 (12.20)		29.70 (28.30)		0.469
Smokers: average (SD)	31.96 (24.60)		33.65 (26.50)		24.67 (15)		0.788
**Alcohol status:** *n* (%)							
Non-drinkers	49 (96.08)		40 (95.24)		9 (100)		1.000
Drinkers	2 (3.92)		2 (4.76)		0		
**Other concomitant diseases:** *n* (%)							
No	12 (23.53)		9 (21.43)		3 (33.33)		0.424
Yes	39 (76.47)		33 (78.57)		6 (66.67)		
Type: *n* (%)							
Cardiopathy	22 (56.41)		17 (40.48)		5 (55.56)		
Thyroid disease	14 (35.90)		12 (28.57)		2 (22.22)		
Diabetes mellitus	2 (5.13)		2 (4.76)		0		
Osteoporosis	11 (28.21)		9 (21.43)		2 (22.22)		
Asthma	1 (2.56)		1 (2.38)		0		
Psoriasis	1 (2.56)		1 (2.38)		0		
Sjogren’s syndrome	1 (2.56)		1 (2.38)		0		
Viral infections (HCV, HIV, and HPV)	4 (10.26)		2 (4.76)		2 (22.22)		
Prostate diseases	2 (5.13)		2 (4.76)		0		
Dyslipidemia	1 (2.56)		1 (2.38)		0		
**Solid or hematological tumor history:** *n* (%)							
No	39 (76.47)		34 (80.95)		5 (55.56)		0.188
Yes	12 (23.53)		8 (19.05)		4 (44.44)		
Type: *n* (%)							
Oral cancer	2 (16.67)		0		2 (22.22)		
Skin cancer	2 (16.67)		1 (2.38)		1 (11.11)		
Breast cancer	2 (16.67)		2 (4.76)		0		
Thyroid cancer	1 (8.33)		1 (2.38)		0		
Esophageal cancer	1 (8.33)		0		1 (11.11)		
Neurofibromatosis	1 (8.33)		1 (2.38)		0		
Kaposi sarcoma	1 (8.33)		1 (2.38)		0		
Melanomas	2 (16.67)		2 (4.76)		0		

Hepatitis C virus (HCV); human immunodeficiency virus (HIV); human papillomavirus (HPV); malignant transformation (MT); standard deviation (SD).

### 3.2. Clinical Characteristics

On average, there were two clinical forms for each patient and approximately four sites. Overall, the most common clinical subtype of OPL was the homogeneous type, followed by the verrucous and speckled types. The analysis revealed that the homogeneous clinical form was more common in patients without MT, and the speckled clinical form was common in patients with MT.

Regarding the site distribution of lesions, the gingiva was the most frequently affected site, followed by the palate and the buccal mucosa. The mean number of involved anatomical sites per patient was 3.59 (±1.65). The percentage of patients with lesions located on the floor of the mouth, the ventral surface of the tongue, and the dorsal surface of the tongue was higher in patients with MT (Table 2). The tongue was the most common site of MT, followed by the gingiva, buccal mucosa, and palate.

### 3.3. Histological Characteristics

The majority of the included patients had approximately three histological features (2.69 ± 0.91). The most common histological features were a band-like lymphocytic infiltrate (92.16%) and a verrucous, nodular, or bulky cellular architecture (41.18%). The verrucous, nodular, or bulky architecture was more common in patients with MT, and the presence of band-like lymphocytic infiltrate was observed in all patients with MT.

The presence of OED was observed in the majority of the included patients (58.82%) and in almost all the patients with MT (88.89%) (Table 3). A statistically significant association was observed between the severity of OED and the risk of transformation, where the increase in the dysplasia grade was strongly correlated with the MT risk (*p* = 0.009). Regarding the histological grading, four patients developed moderately differentiated OSCC (G2), while three patients developed well-differentiated OSCC (G1), and one patient developed a poorly differentiated OSCC (G3).

### 3.4. Management Strategies

The average number of clinical follow-up visits and biopsies, both annually and in total, was slightly elevated in OPL patients with MT, without statistical significance. In the nine OPL patients who experienced MT, the mean duration from the first biopsy to confirmed transformation was 837 days (±1544.68). This large standard deviation was due to some cases exhibiting very long intervals between initial diagnosis and MT.

A total of 30 patients were subjected to surgical treatment. This included laser ablation (60%), excision biopsy (13.33%), and both (26.67%). Among the nine patients who developed MT, six patients were managed conservatively with a “watch-and-wait” approach, while three patients were subjected to a surgical approach: one patient with an excisional biopsy, one with laser ablation, and one with both. The histopathological assessment of the resulting OSCC showed that lesions arising after the conservative approach were predominantly moderately or poorly differentiated, while those developing after surgical treatment were primarily well- or moderately differentiated. These findings suggest a possible trend towards more favorable histological grades in surgically treated patients; however, the small sample size precludes drawing any statistically significant conclusions (Table 4).

## 4. Discussion

This retrospective study investigated the clinical, histopathological, and treatment-related strategies associated with MT in patients diagnosed with OPL. Our findings further suggest that several clinical and pathological variables—i.e., lesion morphology, anatomical site, presence of moderate-to-severe dysplasia, and a history of oral cancer—may influence the risk of malignant progression. These observations are largely consistent with the previously published studies. However, some of our results reflect a lower magnitude of risk, which may be attributed to the characteristics of our patient population, the study design, and the small sample size.

The overall MT rate in our cohort was 17.6%, which is notably lower than the rates reported in OPL studies, where the pooled estimates tend to range between 45% and 61%, with an overall MT rate of 10% annually and mortality rates around 12% [4,5]. Ramos-Garcia et al., in a large meta-analysis, reported a pooled MT rate of 43.87% for OPL, with a wide variation, ranging from 14.3% to 100.0%, depending on the clinical and histological variables. The large variability in the MT rates across the studies was determined by the authors to be due to the clinical heterogeneity, geographical areas, differences in study design, and OPL assessment criteria [15]. Similarly, Lafuente et al. observed that MT rates across studies varied greatly, ranging from 30.0% to 100%. The authors found that higher rates of MT were more frequent in older studies compared to the newer or most recent studies performed in the same medical center and in retrospective studies (after the MT developed), which could represent an important bias [15].

The observed differences may also be influenced by variations in the oral microbiome. In the literature, several systemic comorbidities—such as diabetes, anemia, thyroid disorders, and cardiovascular disease—have been proposed as potential contributors to alterations in the oral environment [16,17]. Emerging evidence further indicates that the oral microbiome may play a role in the malignant transformation of potentially malignant lesions, including OPL. Specific microbial shifts, such as an enrichment of Fusobacterium, Leptotrichia, Campylobacter, and Rothia species, have been reported in oral leukoplakia. Similarly, dysbiosis characterized by reduced microbial diversity and an increased abundance of pathogenic taxa has been associated with both potentially malignant and malignant oral conditions [18,19].

Moreover, emerging genomic and epigenetic evidence is beginning to clarify the molecular mechanisms underlying OPL progression. Recent studies have identified specific alterations—such as distinct DNA methylation patterns, loss of heterozygosity, and recurrent mutations in genes including FAT1, CDKN2A, and NOTCH1 and components of the WNT pathway—that may contribute to the aggressive biological behavior of this condition [20,21,22]. These findings may offer promising avenues for future risk-stratification models. Unfortunately, our retrospective design did not allow for molecular testing. Prospective studies integrating genomic, epigenetic, and clinical parameters are recommended to establish these findings and refine the prediction of MT in OPL [9].

The clinical morphology was reported as a significant determinant of MT. In this study, patients with homogeneous OPL showed a reduced MT risk, while those with speckled lesions showed an increased risk of transformation. This is consistent with previous studies that have consistently shown non-homogeneous leukoplakias—including speckled, verrucous, and erythroleukoplakic types (PEL)—to carry a substantially higher risk than homogeneous lesions. Pimenta-Barros et al. reported a relative risk of approximately 4.2 for non-homogeneous leukoplakias compared to their homogeneous counterparts [1]. Similarly, Villa et al. reported a high prevalence of MT in patients with non-homogeneous OPLs, including patients with erythematous features with an MT of 100% [5]. Our findings corroborate this trend, reinforcing the prognostic value of clinical appearance, which is especially important, as it is immediately assessable in clinical settings without invasive diagnostic procedures.

In this study, the most common sites for MT were the tongue (both ventral and dorsal surfaces), gingiva, buccal mucosa, palate, and floor of the mouth. These findings are consistent with previous reports identifying the lateral tongue, ventral tongue and floor of the mouth as the anatomical locations at highest risk for malignant progression [23,24]. Some authors suggested the presence of specific carcinogenic factors that particularly affect these anatomical sites and favor the MT in OPL patients [24].

Histopathological evaluation further supported the prognostic significance of dysplasia. Although the mere presence of OED was not statistically significant in predicting MT, there was a significant association between the increasing grade of dysplasia and MT. All patients with moderate and severe dysplasia were candidates for MT. This observation is consistent with the reported data in the literature [4,11]. Several studies have consistently shown that the severity of epithelial dysplasia correlates strongly with the risk of malignant progression. This has become a cornerstone in clinical decision-making for the stratification and management of patients with leukoplakia [4,11,25].

In the study, a band-like lymphocytic infiltrate in the histological evaluation was highly frequent in patients with MT. Although there are still unclear data and misconceptions on lichenoid features (LF), as “clinical and histopathological features” in OPL, there are different authors who have reported LF in patients with OPL [6,7]. The specific implications of this inflammatory pattern remain unclear. This inflammatory pattern may represent a non-specific inflammatory response against the leukoplakia or the OED, rather than a true lichenoid lesion [26]. These findings underscore the multifactorial nature of MT risk and highlight the importance of not underestimating oral multisite lesions with clinical and histopathological lichenoid morphology. Lesions with LF may represent a distinct subtype of OPL, rather than being an oral lichenoid lesion (OLL) or a classic oral lichen planus.

Regarding the patient-related risk factors, a higher percentage of the presence of a previous history of solid or hematological tumor was observed in patients with MT. Few studies have focused on this possible variable. In the literature, there is a suggestion to consider patients with a history of head and neck cancer as patients at high risk for developing new primary tumors or experiencing recurrence [27,28,29].

In addition, four patients showed a history of viral infections, and two of them experienced MT. The role of viral infections in OPL has been investigated in the literature because earlier studies suggested the possible presence of a correlation between HPV and OPL [30,31,32]. In a study of a retrospective analysis of 20 years of the association with HPV, no strong evidence was observed linking HPV to a higher rate of MT in OPL patients [32]. A recent systematic review and meta-analysis reported a low prevalence of HPV in OPL and did not highlight HCV or HIV as significant factors in disease development [31].

In our study, one OPL patient with MT had a history of HCV infection. It should be noted that distinguishing between OLL and OPL can be challenging, and the presence of HCV may further complicate this differentiation. Some studies reported a higher prevalence—without robust evidence—of potentially malignant oral disorders, particularly OLL or oral lichen planus, in HCV-infected patients [33,34]. These considerations highlight the need for cautious interpretation of the potential role of HCV in MT within OPL.

Regarding the management strategy (surgical vs. conservative “watch and wait”), no significant association with MT was observed. These findings differ from some previous studies, which reported a reduction in the risk of MT with the surgical removal of leukoplastic lesions, without eliminating the risk for malignant progression, especially in multifocal or field-cancerized contexts such as OPL [35,36].

In a recent randomized clinical trial, Lombardi et al. evaluated the surgical treatment vs. “watch and wait” in patients with homogeneous and non-homogeneous leukoplakia. Although the authors excluded OPL patients with MT, patients in the “watch and wait” group showed a higher frequency of MT compared to patients in the surgical group [35]. In contrast, the MT was evaluated in 260 patients with OL in another trial. The patients were divided into a group subjected to only standard of care and a group subjected to both surgical excision and standard of care. The authors found that one patient in each group experienced MT, and the surgical group showed an inferior outcome with a lower probability of the treated lesions remaining healthy without recurrence. The authors suggested the presence of other underlying factors that affect the MT rate, rather than the kind of management [36]. Other authors attributed this reported absence of difference between “watch and wait” and surgical intervention in the literature to several factors, including limited follow-up frequency, poorly defined lesion margins at excision, variability in clinician experience, and the presence of extensive lesions that require staged removal with clear margins [37,38].

From this experience, some observed limitations should be acknowledged for proper interpretation of the results and consideration in future studies. The first limitation is the study’s single-center design, which limits the generalizability of the findings to larger populations. Consequently, the results cannot be considered fully representative of broader epidemiological data. Second, the inclusion of all potential variables, as well as microbiome and molecular profiling, could not be performed due to the retrospective nature of this study. The inclusion of these assessments in prospective studies may be helpful, as they could provide mechanistic insights, improve risk stratification, decrease possible misdiagnosis bias, and guide individualized surveillance and management of OPL. Third, the study’s sample size was small. Wide confidence intervals for the calculated ORs were observed for several associations, which may be due to this limitation and the low number of events per variable. A multicenter study with a larger sample could yield more definitive results. Fourth, the inclusion of OPL patients was strictly based on recently proposed diagnostic criteria, due to the ongoing debate regarding the diagnosis of OPL. Some probable cases of OPL were excluded because they did not meet these criteria. In addition, the final cohort was limited by the exclusion of all patients who did not complete at least 12 months of follow-up, which substantially reduced the number of eligible OPL cases. This strict criterion was applied to ensure the reliable assessment of MT outcomes.

## 5. Conclusions

In conclusion, this study further suggests that the clinical morphology, lesion site, and histological grading may be important predicting factors for MT in OPL. The presence of a non-homogeneous lesion form, a higher grade of dysplasia, and a history of previous solid or hematological tumor showed a more aggressive disease course. Individualized risk assessment and long-term surveillance may be advisable. Future studies with larger cohorts and longer follow-up periods, as well as the integration of molecular biomarkers, may help to refine risk stratification and guide more personalized management strategies.

## Figures and Tables

**Table 2:** An overview of and comparison between oral proliferative leukoplakia (OPL) patients with and without malignant transformation (MT) according to the clinical characteristics.

Variables	Total (*n* = 51)		Without MT (*n* = 42)		With MT (*n* = 9)		*p*-Value
**Clinical from:** *n* (%)							
Homogeneous	36 (70.59)		33 (78.57)		3 (33.33)		
Verrucous	26 (50.98)		22 (52.38)		4 (44.44)		
Nodular	8 (15.69)		6 (14.29)		2 (22.22)		
Speckled	10 (19.61)		6 (14.29)		4 (44.44)		
**Number of clinical forms:** average (SD)	1.57 (0.78)		1.60 (0.77)		1.44 (0.88)		0.904
**Site distribution of lesions:** *n* (%)							
Buccal mucosa	20 (39.22)		17 (40.48)		3 (33.33)		
Dorsal surface of the tongue	11 (21.57)		7 (16.67)		4 (44.44)		
Lateral borders of the tongue	17 (33.33)		13 (30.95)		4 (44.44)		
Ventral surface of the tongue	14 (27.45)		10 (23.81)		4 (44.44)		
Floor of the mouth	5 (9.80)		2 (4.76)		3 (33.33)		
Alveolar mucosa	14 (27.45)		13 (30.95)		1 (11.11)		
Hard and soft palate	23 (45.10)		21 (50)		2 (22.22)		
Gingiva	37 (72.55)		31 (73.81)		6 (66.67)		
**Number of affected sites:** average (SD)	3.59 (1.65)		3.57 (1.76)		3.67 (1.12)		0.830

Malignant transformation (MT); standard deviation (SD).

**Table 3:** Oral Proliferative Leukoplakia (OPL): Histologic Grading, Sites and Risk of Developing Oral Squamous Cell Carcinoma (OSCC).

Variables	Total (*n* = 51)	Without MT (*n* = 42)	With MT (*n* = 9)	*p*-Value
**Histological features:** *n* (%)				
HkNR	21 (41.18)	20 (47.62)	1 (11.11)	
Epithelial hyperplasia	17 (33.33)	13 (30.95)	4 (44.44)	
Band-like lymphohistiocytic infiltrate	47 (92.16)	37 (88.10)	9 (100)	
Verrucous, nodular, or bulky architecture	21 (41.18)	16 (38.10)	6 (66.67)	
**Number of histological features:** average (SD)	2.69 (0.91)	2.60 (0.89)	3.11 (0.93)	0.149
**Presence of dysplasia:** *n* (%)				
No	21 (41.18)	20 (47.62)	1 (11.11)	0.064
Yes	30 (58.82)	22 (52.38)	8 (88.89)	
**Highest grade of dysplasia:** *n* (%)				
Low	26 (86.67)	22 (100)	4 (50)	**0.009**
Moderate	3 (10)	0	3 (37.5)	
High	1 (3.33)	0	1 (12.5)	
**Malignant transformation:** *n* (%)				
Yes	9 (17.65)			
No	42 (82.35)			
Duration from 1° biopsy and malignant transformation (days): average (SD)	837 (1544.68)			
**Histological grading:** *n* (%)				
In situ	1 (11.11)			
G1	3 (33.33)			
G2	4 (44.44)			
G3	1 (11.11)			
**Site of OSCC:** *n* (%)				
Tongue (2B62)	5 (55.56)			
Gingiva (2B63)	3 (33.33)			
Buccal mucosa (2B66)	3 (33.33)			
Palate (2B65)	1 (11.11)			

Hyperkeratosis non-reactive (HkNR); malignant transformation (MT); oral squamous cell carcinoma (OSCC); standard deviation (SD).

**Table 4:** An overview and comparison between oral proliferative leukoplakia (OPL) patients with and without malignant transformation (MT) according to the management strategies.

Variables	Total (*n* = 51)		Without MT (*n* = 42)		With MT (*n* = 9)		*p*-Value
**Number of follow-ups per year:** average (SD)	4.82 (1.80)		4.69 (1.72)		5.44 (2.13)		0.337
**Number of performed biopsies:** average (SD)	3.98 (2.86)		3.60 (2.52)		5.78 (3.77)		0.078
**Number of performed biopsies per year:** average (SD)	1.05 (0.89)		0.96 (0.86)		1.43 (1.00)		0.104
**Number of years of follow-up:** average (SD)	4.47 (2.45)		4.40 (2.45)		4.78 (2.59)		0.764
**Management protocols:** *n* (%)							
Watch and wait	21 (41.18)		15 (35.71)		6 (66.67)		0.136
Surgical treatment	30 (58.82)		27 (64.29)		3 (33.33)		
Type: *n* (%)							
Laser ablation	18 (60.00)		17 (62.96)		1 (33.33)		
Excision biopsy	4 (13.33)		3 (11.11)		1 (33.33)		
Both	8 (26.67)		7 (25.93)		1 (33.33)		

Malignant transformation (MT); standard deviation (SD).

## Data Availability

The datasets used and/or analyzed during the current study are available from the authors on reasonable request.

## Supplementary Materials

The following supporting information can be downloaded at https://www.mdpi.com/article/10.3390/cancers18010002/s1, Table S1: Oral proliferative leukoplakia (OPL) characteristics, according to the proposed criteria by Villa et al. [5]; Table S2: Odds ratios (ORs) and their 95% confidence intervals (CIs) for all the study variables.

## Abbreviations

The following abbreviations are used in this manuscript:

OPL	Oral proliferative leukoplakia
MT	Malignant transformation
OL	Oral leukoplakia
WHO	World Health Organization
OED	Oral epithelial dysplasia
OSCC	Oral squamous cell carcinoma
OVC	Oral verrucous carcinoma
ICD	International Classification of Diseases
PEL	Proliferative erythroleukoplakia
OLL	Oral lichenoid lesions
