# Supplementary material for: Risk Assessment for Malignant Transformation in Patients with Oral Proliferative Leukoplakia: A 10-Year Retrospective Cohort Study
Source: Cancers (Basel). 2025 Dec 19;18(1):2. doi: 10.3390/cancers18010002 (PMC12785022; doi:10.3390/cancers18010002)
Supplement: Supplementary file 1 [file cancers-18-00002-s001.zip › cancers-3962212-supplementary.pdf]

Table S1. Oral proliferative leukoplakia (OPL) characteristics according to the proposed criteria by Villa et al. [5].

**Clinical aspect**	- Presence of white or keratotic lesions.<br>- The lesions can be smooth, fissured, verrucous, and/or erythematous with or without ulceration.
**Extent and multifocality**	They can be presented as:<br>- Discontinuous lesions distributed across multiple sites of the oral mucosa,<br>- Single lesions of significant size (greater than 4 cm if limited to a single site, or greater than 3 cm if extended to adjacent sites).
**Progression**	- Lesions must demonstrate an expansive behavior over time, with a tendency to enlarge and/or develop new, multifocal foci.
**Histological aspect**	- Presence of overt dysplasia or carcinoma.<br>- Alternatively, the microscopic picture must show alterations such as hyperkeratosis, parakeratosis, atrophy, or acanthosis, with no or minimal cytological atypia.<br>- These findings may possibly include verrucous hyperplasia or a subepithelial lymphocytic band, but they should not be consistent with a diagnosis of frictional keratoses or reactive keratoses.

Table S2. Odds ratios (ORs) and their 95% confidence intervals (CIs) for all the study variables.

Variables		OR (95% CI)
**Demographic and anamnestic data**		
**Age:**		
	≤60	1
	>60	0.40 (0.0777 - 2.27)
**Gender:**		
	Female	1
	Male	0.625 (0.145 - 2.70)
**Tobacco status:**		
	Non-smokers	0.667 (0.147 - 3.03)
	Ex-smokers vs.	0.710 (0.151 - 3.33)
	Smokers	1.12 (0.241 - 5.16)
**Other concomitant diseases:**		
	Yes	0.545 (0.114 - 2.62)
	No	1
**Solid or hematological Tumor history:**		
	Yes	1
	No	2.940 (0.064 - 1.350)
**Histological characteristics**		
**Presence of dysplasia:**		
	Yes	0.138 (0.015 - 1.20)
	No	**1**
**Management strategies**		
**Management:**		
	Surgical treatment	3.60 (0.785 - 16.5)
	Watch and wait	1

Confidence intervals (CI); malignant transformation (MT); odd ratio (OR)
